# Superposition of AC-DBD plasma actuator outputs for three-dimensional disturbance production in shear flows

**DOI:** 10.1007/s00348-023-03616-9

**Published:** 2023-04-11

**Authors:** John W. Kurelek, Marios Kotsonis, Serhiy Yarusevych

**Affiliations:** 1 Department of Mechanical and Mechatronics Engineering, University of Waterloo, Waterloo, ON Canada; 2 Faculty of Aerospace Engineering, Delft University of Technology, Delft, The Netherlands

## Abstract

This investigation explores the utility of Alternating Current, Dielectric Barrier Discharge (AC-DBD) plasma actuators for producing three-dimensional disturbances of a desired spanwise wavelength via superposition. The technique utilizes two pairs of exposed and covered electrodes on a single dielectric layer arranged in streamwise succession. Two-dimensional forcing is achieved through operation of the upstream, spanwise uniform electrode pair, while three-dimensional forcing at a prescribed spanwise wavelength is attained by operating both electrode pairs simultaneously, with the downstream actuator spanwise modulating the upstream, two-dimensional output. The ability to produce disturbances of different spanwise wavelengths but with equal streamwise wavelength, frequency and total momentum is established through a combined characterization effort that considers quiescent and in-flow conditions. A demonstration of the technique in an exemplary wall-bounded shear flow, a laminar separation bubble, is provided, revealing spanwise wavelength dependent disturbance growth in the flow that could be exploited for performance gains in future flow control endeavours.

**Graphical abstract:**

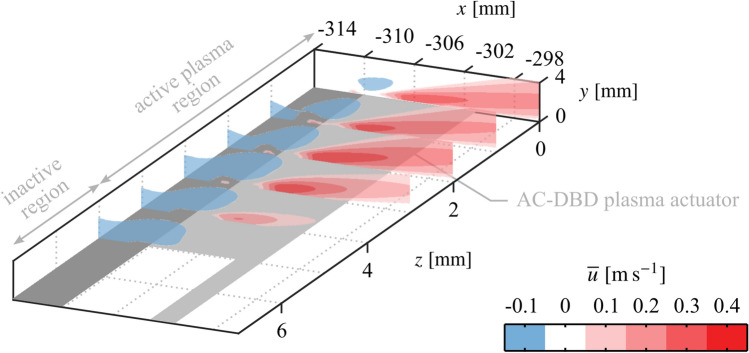

**Electronic supplementary material:**

The online version contains supplementary material available at (10.1007/s00348-023-03616-9).

## Introduction

Plasma actuators have seen a continued rise in popularity as aerodynamic flow control devices over the last three decades (Corke et al. 2010). This can be attributed to an attractive set of features compared to other active flow control techniques that includes a lack of moving parts and a relatively broad frequency-response bandwidth that extends into the kilohertz range with no resonance restrictions. Among different types of plasma actuators, AC-DBD actuators are the most commonly used in aerodynamic flow control applications. For example, plasma actuators have seen use in studies aimed at separation control (Post and Corke 2004; Huang et al. 2006; Sato et al. 2019), generating coherent structures (Jukes and Choi 2013; Weingaertner et al. 2020), turbulent boundary layer conditioning (Jacob et al. 2004; Schatzman and Thomas 2010), transition delay (Grundmann and Tropea 2008; Kotsonis et al. 2013), and bluff body wake reduction (Thomas et al. 2008; Kelley et al. 2014). An array of past and potential applications of plasma actuators for flow control is discussed in topical reviews by Moreau (2007) and Corke et al. (2010), and comparisons to other types of flow actuators can be found in Cattafesta and Sheplak (2011).

**Fig. 1:**
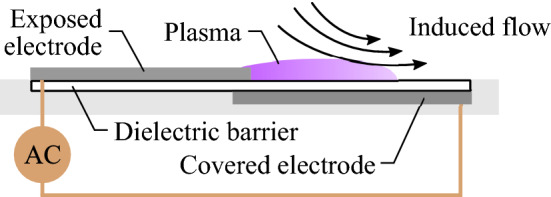
Typical asymmetric AC-DBD plasma actuator configuration

A typical asymmetric AC-DBD plasma actuator configuration is depicted in Fig. 1, where plasma generation leads to momentum exchange with the surrounding air through ion-neutral collisions, with the asymmetry of the electrodes and the presence of a dielectric layer leading to unequal amounts of momentum transferred during the two AC half cycles. A net body force is generated, giving rise to a relatively weak and nearly wall-parallel jet (e.g., Kotsonis and Ghaemi 2011). Common electrode materials include adhesive-backed copper or conductive paint, while polyimide (Kapton) tape, polyethylene terephthalate (PET), or acrylic are often used for the dielectric layer (Forte et al. 2007). The electrodes are fixed to the dielectric either by hand or through CNC deposition, with final thicknesses typically kept under 1 mm to allow for integration into test models (Corke et al. 2010).

The most widely studied DBD configuration is the linear actuator design, where the overlap between the electrodes, i.e., the discharge line, is continuous and uniform across the span of the device. When oriented parallel to the incoming flow, a continuous or pulsed spanwise uniform front can be produced, both of which have been shown to be effective at maintaining and/or promoting spanwise uniformity in nominally two-dimensional flows (e.g., Thomas et al. 2008; Grundmann and Tropea 2008; Sato et al. 2019). Alternatively, streamwise-oriented coherent structures can be generated by orienting the discharge line longitudinally, as demonstrated by Jukes and Choi (2013), which investigators have used for separation control in place of physical vortex generators (e.g., Vernet et al. 2018), and for streak cancellation in transitional boundary layers (e.g., Hanson et al. 2010).

The ability to rapidly prototype devices has led investigators to develop and test more exotic configurations aimed at specific flow control objectives. Examples include wedge and v-shaped actuators for vectoring the plasma induced jet (Porter et al. 2009), annular and counter-flow arrangements that produce wall-normal jets (Santhanakrishnan et al. 2006; Mishra et al. 2022), and configurations that utilize multiple electrodes to increase total momentum output, e.g., the ‘trielectrode sliding’ arrangement of Moreau et al. (2008). Furthermore, various configurations have been developed to produce spanwise non-uniform disturbance fronts, such as sinusoid/serpentine-shaped (Durscher and Roy 2012b; Mohammadpour et al. 2021), horseshoe-shaped (Roy and Wang 2009) and spanwise segmented actuators for cross-flow instability control (Serpieri et al. 2017; Yadala et al. 2018).

The ability to produce flow disturbances modulated to a specific spanwise wavelength has long been a topic of interest within the fluid mechanics research community, since such tools have proven useful in elucidating key physical mechanisms in several canonical flows. Perhaps most well-known is the study of Klebanoff et al. (1962), who introduced three-dimensional disturbances into a laminar boundary layer by spanwise-modulating the output of a thin vibrating ribbon, showing ensuing spanwise deformations of Tollmien-Schlichting waves into three-dimensional structures. Since, researchers have adopted this approach in boundary layers studies (Saric et al. 2003), in addition to free shear layers (e.g., Wu et al. 1993) and laminar separation bubbles (e.g., Marxen et al. 2004). However, most of such studies were numerical simulations, where implementing deterministic, three-dimensional input disturbances is more straightforward compared to experiments.

A laminar separation bubble (LSB) is one case in particular where controlled three-dimensional perturbations have been considered and can aid in understanding the complex physics in the attendant flow development. LSBs are common on airfoils operating at aerodynamically low Reynolds numbers (Lissaman 1983), where they form due to laminar boundary layer separation and subsequent mean flow reattachment driven by shear layer transition (e.g., Gaster 1967). A relatively rapid amplification of perturbations in the separated shear layer leads to vortex shedding, with the formed shear layer vortices developing spanwise deformations prior to the breakdown to turbulence (Burgmann and Schröder 2008; Jones et al. 2008; Hain et al. 2009; Michelis et al. 2018; Kurelek et al. 2020). This points to the possibility of an underlying spanwise instability that numerical studies have shown to be active using deterministic disturbance inputs (Rist and Augustin 2006; Marxen et al. 2013). However, a definitive experimental validation and investigation of the development of different instability modes are impeded by the difficulty in implementing a practical and reliable spanwise modulated forcing technique. Furthermore, there is significant impetus to develop such a technique since inducing mean flow reattachment on a stalled airfoil (and thus forming an LSB) or reducing the size of an existing LSB is most effective when forcing targets the shear layer instability (Marxen and Henningson 2011; Yarusevych and Kotsonis 2017). However, most techniques increase the spanwise coherence of the shear vortices which exacerbates airfoil self-noise generation; a phenomenon that occurs when strongly periodic and spanwise-coherent structures pass over the airfoil trailing edge (e.g., Desquesnes et al. 2007; Pröbsting and Yarusevych 2015). Thus, a technique that simultaneously gains control authority over the LSB but also reduces the spanwise coherence of its shear layer vortices, e.g., through spanwise modulated forcing actions, could be highly desirable in such circumstances.

Based on previous studies, it is clear that the approach of introducing controlled perturbations to a flow and studying its response has proven to be an invaluable tool toward advancing our fundamental understanding of fluid flows, which began in the experimental domain but has continually shifted to simulations. The advent of plasma actuators as aerodynamic flow control devices presents an opportunity for renewed experimental work in this area given the versatility of these devices. This, coupled with an opportunity for real-world application, motivates the present study to develop a new forcing technique capable of producing three-dimensional disturbance at a prescribed spanwise wavelength using plasma actuators. The approach involves spatial superposition of disturbances produced by a set of AC-DBD actuators, with a full characterization of the technique accomplished using Particle Image Velocimetry (PIV).

## Experimental setup

**Fig. 2:**
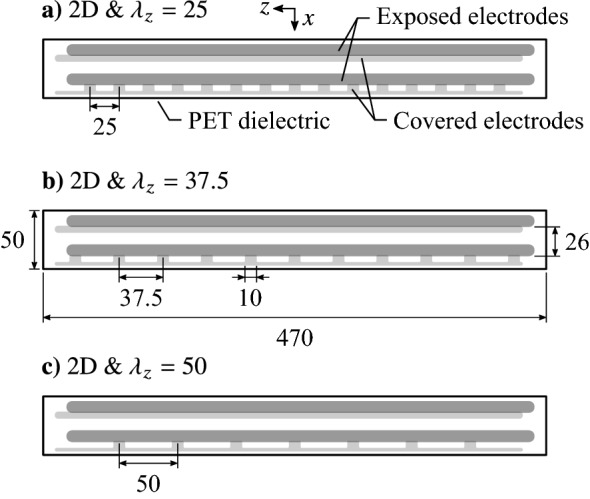
Plasma actuator configurations. All dimensions in millimetres

The AC-DBD plasma actuators used in this investigation are shown Fig. 2. Each device consists of two pairs of exposed and covered electrodes on the same dielectric. As will be demonstrated, during operation a weak wall-parallel jet near the surface is produced in the regions where electrode pairs overlap. A 400 $\upmu$m thick PET strip served as the dielectric layer, onto which the electrodes were painted using conductive silver paint. Each painted electrode was approximately 5 $\upmu$m thick, making the total thickness of the actuator 410 $\upmu$m. The actuators were flush mounted in appropriately sized grooves machined into the test model. The exposed and covered electrodes extended 10 and 6 mm in the streamwise direction, *x*, respectively, and were overlapped by 1 mm.

Three-dimensional disturbances were produced by arranging the electrode pairs in streamwise succession (Fig. 2), with the upstream pair used to produce a two-dimensional disturbance, while the downstream pair imposed a spanwise modulation as a result of gaps in the covered electrode in the *z*-direction. Three actuator configurations were fabricated with spanwise wavelengths of $\lambda _{z}= 25$, 37.5, and 50 mm. Four forcing configurations are considered, the first consisting of pure two-dimensional forcing achieved through operation of only the upstream set of electrodes. The other three cases are three-dimensional forcing at spanwise wavelength of $\lambda _{z}= 25$, 37.5, and 50 mm, with each achieved by operating both sets of electrodes simultaneously with a relative phase delay used to superimpose the disturbances introduced into the flow.

The influence of external flow conditions on AC-DBD actuator output presents challenges for actuator characterization (Pavon et al. 2007; Kriegseis et al. 2012; Kotsonis 2015), particularly when knowledge of both momentum output and produced disturbance characteristics are desired. Pereira et al. (2014) note that in low-speed flow applications, actuator momentum output is largely insensitive to external flow conditions, and is therefore amenable to quiescent characterization. As such, the approach to actuator characterization is divided between a quiescent characterization of momentum output (Sect. 2.1) and an in-flow characterization of flow disturbances where conditions are matched to an exemplary application of interest (Sect. 2.2).

The electrode pairs were driven independently by two TREK 20/20C high voltage amplifiers with signal generated using National Instrument’s LabVIEW software and an NI 9260 analog output module. The forcing signals consisted of a $f_\text {c}$ = 5 kHz carrier sine wave modulated by a $f_\text {m}$ = 133 Hz square wave and amplified to a peak-to-peak voltage of $V_\text {pp}$ = 6 kV. For these forcing parameters, the effects of viscosity are expected to be significant in quiescent conditions given the relatively low velocities generated by the actuator (on the order of $1\, \text {ms}^{-1}$). This results in low levels of turbulent mixing and an uneven distribution of seeding particles for PIV measurements, with particularly low particle density found in the ionization region (i.e., the region of interest). This is exacerbated at low duty cycle actuation, making it necessary to operate the actuator at higher duty cycles to enable sufficient mixing of seeding particles, and then scale the momentum output accordingly. Such an approach is feasible due to the large separation of characteristic time scales between the fluid and plasma dynamics, effectively decoupling the associated phenomena (Jayaraman et al. 2007). Therefore, the modulation duty cycle was varied between 18% and 100% for quiescent characterization to establish the relationship between duty cycle and momentum output, with values ranging between 21% and 25% selected for in-flow characterization.

### Quiescent characterization

**Fig. 3:**
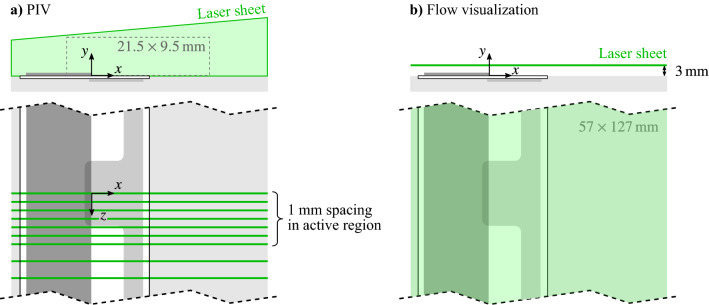
Experimental setup for actuator characterization in quiescent conditions. **a** PIV measurements and **b** flow visualizations. Actuator thickness (*y*-height) not to scale

The experimental setup for quiescent characterization is shown in Fig. 3. Each electrode pair was characterized individually, with flow visualizations and planar PIV measurements conducted in the region of electrode overlap, where ionization occurs and the wall jet is produced. The measurements were carried out in quiescent conditions created within a contained volume with dimensions $610 \times 610 \times 2400$ mm, with the actuator mounted flush to the surface of a $250 \times 6$ mm ABS plate. The volume was seeded by atomizing olive oil into particles with a mean diameter on the order of 1 $\upmu$m using a Laskin nozzle style atomizer based on the designs of Kähler et al. (2002). Olive oil was selected in place of water-based particles due to the significant electrodynamic effects that prevent substances with strongly polar molecules from entering the ionization region (Durscher and Roy 2012a; Kotsonis 2015).

Particle image acquisition for both the flow visualizations and PIV measurements was carried out using an EverGreen 70 mJ/pusle Nd:YAG laser, a LaVision Imager Pro-X 2M camera, a timing unit, and LaVision’s DaVis software. The flow visualizations (Fig. 3b were completed in a top view orientation, with the laser beam conditioned into a 2 mm thick sheet that was positioned in an *x*-*z* plane centred 3 mm from the surface. The camera was fitted with a 60 mm focal length Nikon macro lens set to $f_\# = 5.6$, its sensor cropped to $1600 \times 711$ px from its full resolution of $1600 \times 1200$ px. The imaged field of view was $127 \times 57$ mm, corresponding to a magnification factor of 0.09.

The PIV measurements (Fig. 3a were completed in a side view orientation, with measurements performed in multiple *x*-*y* planes. The camera and laser were moved in tandem using an automating traversing system, with calibration images taken at multiple planes ensuring positional accuracy within ±0.25 mm. The laser sheet thickness and camera resolution were maintained from the flow visualizations, while the camera was fitted with a 200 mm focal length Nikon macro lens set to $f_\# = 8$. The field of view was $21.5 \times 9.5$ mm, corresponding to a magnification factor of 0.55. The images were acquired in double-frame mode, with 2000 images pairs recorded per measurement plane at 14.67 Hz. The frame separation time was set to 800 $\upmu$m, which kept particle displacements under 15 px. Velocity fields were computed using an iterative, multi-grid cross-correlation scheme with window deformation (Scarano and Riethmuller 2000), with a final window size of $24 \times 24$ px (75% overlap). The vector pitch in the PIV data is 0.08 mm, with results post-processed using the universal outlier detection algorithm (Westerweel and Scarano 2005). Based on the correlation statistics method (Wieneke 2015), the average uncertainty in the core of the actuator wall jet region is estimated to be less than 3.2%, while higher uncertainties are generally present in the ionization region due to the aforementioned low levels of turbulent mixing and hence uneven seeding distribution in this area.

### In-flow characterization

**Fig. 4:**
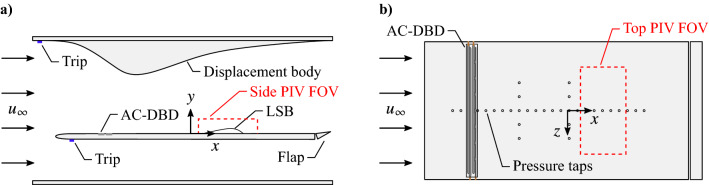
**a** Side and **b** top view of experimental setup for in-flow actuator characterization

The in-flow characterization was conducted at Delft University of Technology in the Anechoic Vertical Low Turbulence Wind Tunnel (A-Tunnel), which is an open-jet, closed-circuit, vertical wind tunnel. The wind tunnel test section measures $500 \times 500 \times 1100$ mm, and features a turbulence intensity of $0.09\%$, a free-stream uniformity within $\pm 1.0\%$, and no significant spectral peaks in the free-stream velocity and pressure fluctuations within $1 \le f \le$ 1000 Hz at the investigated free-stream velocity, $u_\infty = 5.75 \, \text {ms}^{-1}$ (Merino-Martínez et al. 2020). A schematic of the experimental setup is provided in Fig. 4, showing the flat plate test model ($1000 \times 500 \times 20$ mm). The model has a super elliptical leading edge (Lin et al. 1992), ensuring seamless curvature change and the development of an attached laminar boundary layer on the top surface at appropriate Reynolds numbers. The trailing edge forms part of an adjustable flap, which was deflected upwards to mitigate possible unsteady leading edge separation effects.

The flow of interest for disturbance characterization is a laminar separation bubble, induced on the flat plate by an applied adverse pressure gradient. An adjustable displacement body on the top wall of the test section provided the adverse pressure gradient, while its boundary layer was tripped by zig-zag type turbulators in order to avoid flow separation on the body (Fig. 4a). The same technique was used to trip the boundary layer on the plate bottom surface to avoid possible vortex shedding at the trailing edge. The Cartesian coordinate system origin was set such that $x = 0$ is the location of mean flow separation with no forcing (490.5 mm from the leading edge).

**Fig. 5:**
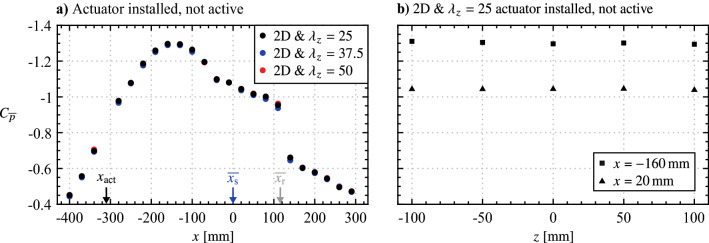
Mean surface **a** streamwise and **b** spanwise static pressure distributions with actuator installed but not active. Measurement uncertainty is given by the marker size. Two-dimensional discharge line of actuator is located at $x_\text {act}$. Blue and grey arrows mark approximate mean streamwise locations of separation ($\overline{x_\text {s}}$) and reattachment ($\overline{x_\text {r}}$), respectively

Integrated into the model are 30 static pressure taps (0.4 mm diameter), 22 of which are arranged in a streamwise row at $z = 0$, with the remaining 8 arranged in two spanwise rows at $x = -160$ and 20 mm. Static pressures from all taps were measured simultaneously at 100 Hz for 20 s using a set of Honeywell HSC series differential pressure transducers with a full range of 160 Pa. Measured pressure distributions, expressed in terms of $C_{{\overline{p}}} = \left( {\overline{p}} - p_\infty \right) /\left( \frac{1}{2}\rho {u_\infty }^2\right)$, are shown in Fig. 5, where the estimated uncertainty for all measurements is $\pm 4.6\%$ of the free-stream dynamic pressure. As indicated by the pressure plateau region in the streamwise pressure distribution (black markers in Fig. 5a), a separation bubble is present with mean separation and reattachment locations of approximately ${\overline{x_\text {s}}} = 0$ and ${\overline{x_\text {r}}}$ = 112 mm, respectively. The spanwise pressure distributions (Fig. 5b) indicate that the flow is essentially two-dimensional, with spanwise uniformity falling within $\pm 0.8\%$.

With the three AC-DBD actuators shown in Fig. 2, a total of five cases were investigated, namely, (i) no forcing (actuator installed but not active), (ii) two-dimensional forcing (only upstream electrode pair active), and three-dimensional forcing with spanwise wavelengths of (iii) 25, (iv) 37.5 and (v) 50 mm. Figure 5a confirms that there is no change in the mean streamwise extent of the LSB, within the experimental uncertainty, as a result of physically changing the actuator device. All actuators were placed to locate the two-dimensional discharge line at $x_\text {act}$= -310 mm.

**Table 1:** PIV parameters for in-flow characterization

Parameter	Side view	Top view	Unit
Laser	Continuum 523-120 M Nd:YAG		–
Camera	PCO Dimax HS4		–
Lens focal length	105		mm
Lens $f_\#$	5.6	2.8	–
Magnification factor	0.28	0.14	–
Sensor resolution	$2016 \times 432$	$1680 \times 848$	px
Total field of view	$79 \times 16$	$135 \times 63$	mm
PIV mode	Double-frame		–
Sampling rate	2	1.75	kHz
Laser pulse sep.	80	140	$\upmu$m
Max. particle disp.	15	12	px
No. of samples	7238	4480	–
Final window size	$24 \times 24$ (75% overlap)		px
Vector pitch	0.24	0.48	mm
Avg. uncertainty	3.5	5.2	% of $u_\infty$

Streamwise and spanwise aspects of the flow development were assessed using planar, time-resolved PIV in side and top view orientations, respectively. Approximate fields of view for these setups are depicted in Fig. 4, and an overview of the PIV parameters is provided in Fig. 1. The flow was seeded using a water-glycol based fog with a mean particle diameter of 1 $\upmu$m produced by a SAFEX generator. Illumination was provided by a Continuum 532-210M Nd:YAG high-speed laser, with the beam conditioned into a sheet approximately 2 mm thick. Images were captured by a PCO Dimax HS4 camera fitted with a 105 mm macro lens set to $f_\# = 5.6$ and 2.8 for the side and top views, respectively. The camera was synchronized with the laser via a LaVision timing unit and image acquisition was performed in LaVision’s DaVis 8 software.

For the side view setup, measurements were taken at multiple *x*-*y* planes so that the flow field could be volumetrically reconstructed using phase-averaging. At each plane, the full camera resolution of $2016 \times 2016$ px was cropped to $2016 \times 423$ px to cover an area of $79 \times 16$ mm, resulting in a magnification factor of 0.28. For the top view setup, measurements were performed at a *x*-*z* plane located 7 mm from the top surface of the plate. In order to achieve the desired sampling rate, the camera’s sensor was cropped to ${1680} \times 848$ px, covering a field of view of $135 \times 63$ mm at a magnification factor of 0.14. Sampling was performed at 2 and 1.75 kHz for a total of 7328 and 4480 samples for the side and top view configurations, respectively. Prior to amplification, the plasma forcing signal was split and sent to the PIV timing unit, allowing for the phase information between the forcing and PIV acquisitions to be determined.

For both PIV setups, the focus was adjusted to produce imaged particles approximately 3 to 4 px in diameter. An iterative, multi-grid cross-correlation scheme with window deformation (Scarano and Riethmuller 2000) was used to compute velocity fields. A final interrogation window size of $24 \times 24$ px with 75% overlap was used, with windows of this size containing, on average, 12 particles. The resulting vector pitches in the data are 0.24 and 0.48 mm for the side and top view configurations, respectively. The results were post-processed using the universal outlier detection method (Westerweel and Scarano 2005). Using the correlation statistics method (Wieneke 2015), the average uncertainties within the region of the separated shear layer are estimated to be less than 3.5% and 5.2% of $u_\infty$ for the side and top view configurations, respectively, while higher uncertainties are present near the wall for the side view configuration.

## Results

Across the three actuator configurations (Fig. 2), four forcing scenarios are considered, including two-dimensional forcing, achieved through operation of only the upstream set of electrodes, and three-dimensional forcing at spanwise wavelengths of $\lambda _{z}= 25$, 37.5, and 50 mm, each achieved by operating both sets of electrodes on a given dielectric simultaneously. Discussion of results begins with quiescent characterization of momentum output for each electrode pair (Sect. 3.1), where parameters at which all four forcing scenarios provide an equal amount of total momentum output are identified. Afterwards, focus shifts to an in-flow characterization (Sect. 3.2), where the phase delay between actuator pairs needed to spatially superimpose their outputs is determined, with the resulting disturbances assessed to determine if the technique is successful in creating perturbations of a desired spanwise wavelength. Lastly, a look at the the effects of the forcing on the flow of choice—a laminar separation bubble—is presented in Sect.3.3.

### Quiescent characterization

The characteristics of the employed plasma actuators (Fig. 2) in terms of basic operation and net momentum injection are discussed in this section. Since these characteristics have been shown to be invariant to external flow conditions in the low subsonic regime (Pereira et al. 2014), the associated experiments are conducted in quiescent conditions.

**Fig. 6:**
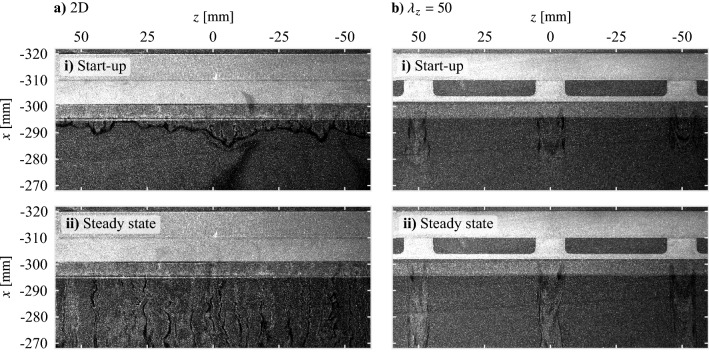
Visualization of flow induced due to plasma actuation during the **i** start-up and **ii** steady state phases ($V_\text {pp}$ = 6 kV, $f_\text {c}$ = 5 kHz, 100% duty cycle). Flow is from top-to-bottom. See supplementary material for accompanying videos (Online Resources 1 and 2, 10.1007/s00348-023-03616-9)

Figure 6 and the associated supplementary videos (Online Resources 1 and 2, 10.1007/s00348-023-03616-9) show visualizations of the flow induced by plasma actuation for the two-dimensional and $\lambda _{z}=50$ mm electrode pairs. The electrode overlap is located at $x = -310$ mm with the exposed and covered electrodes located at $x < -310$ mm and $x > -310$ mm, respectively. The induced jets are visible in the images due to the non-uniform dispersion of seeding particles in regions of high shear, which reveals that the jets are aligned in the streamwise direction and appear downstream of the electrode overlap location. In Fig. 6a–i, the output of the two-dimensional configuration is spanwise non-uniform during start-up, showing similarities to the spanwise wavy plasma discharge front found by Benard et al. (2012), which is promptly followed by the steady-state operation to form a spanwise-uniform discharge (Fig. 6a–ii).

For the $\lambda _{z}$= 50 mm actuator, Fig. 6b reveals this configuration only induces flow within the regions of electrode overlap, i.e., the active regions, with the flow remaining essentially stagnant in between. Flow visualizations for the other electrode pairs (not shown for brevity) show similar results in Fig. 6b, confirming a successful design as the flow is forced in a spanwise modulated manner at the intended wavelength. Of particular note, no inter-electrode plasma forms between adjacent active regions since all spanwise wavelengths exceed the plasma discharge length, estimated to be on the order of 1 mm based on the maximum forcing voltage (3 kV, 6 kV peak-to-peak) and the typical breakdown voltage of dry air at atmospheric pressure (30 kV cm^−1^, Rigden 1996). The latter estimate can serve as an approximate limit for the minimum feasible spanwise forcing wavelength, which depends on the operating parameters.

**Fig. 7:**
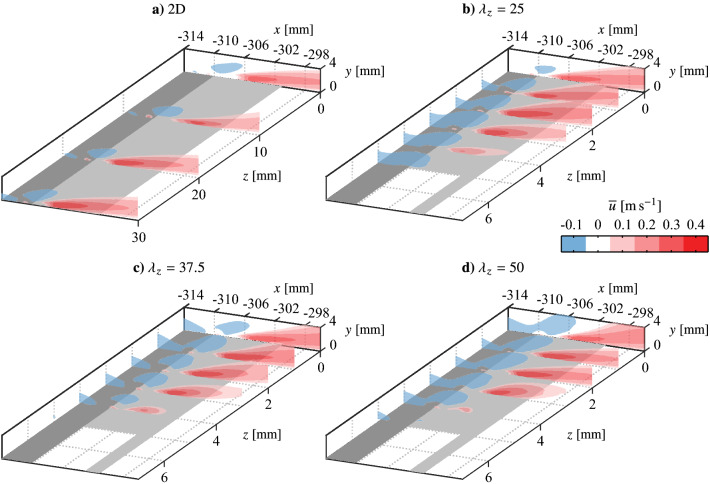
Contours of mean streamwise velocity across actuator spans ($V_\text {pp} ={6}$ kV, $f_\text {c} = {5}$ kHz, 100% duty cycle). Note different aspect ratios between **a** and **b**–**d**

PIV measurements are used to quantify the flow induced by the actuator and estimate the momentum imparted to the fluid. Figure 7 presents contours of mean streamwise velocity across the span of the four electrode pair configurations. As is typically seen for surface mounted AC-DBD plasma actuators, at active locations sufficiently removed from end effects (e.g., *z* = 0 mm), all configurations produce a wall jet predominantly in the streamwise direction with a minor inclination angle relative to the surface that originates just downstream of the electrode overlap (*x* = − 310 mm). For the two-dimensional configuration (Fig. 7a), consistent with the flow visualizations (Fig. 6a), good spanwise uniformity is seen across the four measurement planes. For the spanwise modulated configurations (Fig. 7b–d), the jet exhibits strong spanwise uniformity near the centre of the active region ($0 \le z \le$ 2 mm), while the jet’s streamwise velocity magnitude decreases as the edge is approached ($2 < z \le$ 4 mm). Measurements at the edge of the actuator overlap (*z* = 5 mm) and in the gap region ($z>$ 5 mm) reveal no positive streamwise velocity at these locations. These observations are consistent with the flow visualizations, showing that the produced jets are more narrow than the active electrode regions and are separated by stagnant fluid. Upon close inspection of the *z* = 4 mm plane in Fig. 7b–d, there is a clear decrease in jet streamwise velocity with increasing spanwise wavelength, which may be the result of a weak interaction effect between adjacent jets. Overall the effect is weak, but it will result in a minor dependence of the total imparted momentum on spanwise wavelength.

**Fig. 8:**
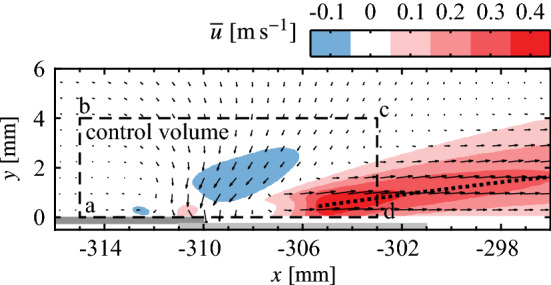
Contours of mean streamwise velocity at *z* = 0 mm for the 2D actuator ($V_\text {pp}$ = 6 kV, $f_\text {c}$ = 5 kHz, 100% duty cycle). Dashed outline (corners abcd) indicates control volume for momentum analysis. Dotted line indicates locus of local mean velocity maxima, ${\overline{u}}_\text {m}$

A control volume analysis is employed to quantify the momentum imparted to the fluid. The selected control volume is shown in Fig. 8, which is used for all configurations. It is noted that the use of planar PIV measurements does not provide a measure of the spanwise transport of momentum, however, the flow visualizations (Fig. 6) provide qualitative evidence that this component is not significant, even for the spanwise modulated configurations. The control volume boundaries are selected to be sufficiently removed from the strong pressure gradients near the electrode overlap (Kotsonis et al. 2011), since an estimate of pressure is not available. Performing a momentum balance in the *x*-direction gives

1 $$\begin{aligned} T_x = \rho \left[ \int _{\text {ab}} {\overline{u}}^2\textrm{d}y + \int _{\text {bc}} {\overline{u}}\,{\overline{v}}\textrm{d}x - \int _{\text {cd}} {\overline{u}}^2\textrm{d}y\right] , \end{aligned}$$

where $T_x$ captures contributions from the streamwise body force applied to the fluid and the wall shear stress. Separation of these two is not possible given the experimental limitations, however, this is not critical given that the objective is quantification of the differences in generated forces between the different configurations. Therefore, $T_x$ provides a suitable approximation of the total thrust force applied to the fluid.

**Fig. 9:**
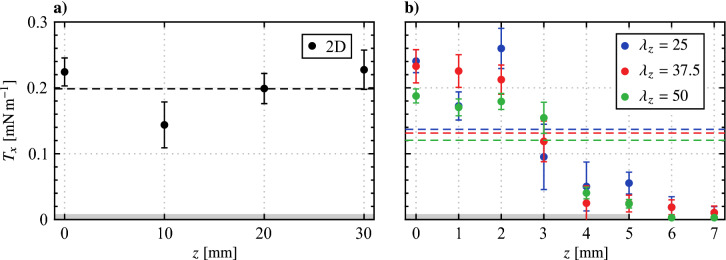
Sectional thrust generated across actuator spans ($V_\text {pp}$ = 6 kV, $f_\text {c}$ = 5 kHz, 100% duty cycle). Actuators are active within the grey shaded regions. Dashed lines (coloured according to legend) indicate average thrust within the active regions, $T_\text {act}$

The results of the momentum calculations are presented in Fig. 9, showing thrust values between $0.14 \le T_x \le$ 0.25 mN m^−1^ are produced by the two-dimensional configuration (Fig. 9a) and within the $0 \le z \le$ 2 mm region for the spanwise modulated configurations (Fig. 9b). These values are expected to be more or less equivalent given that thrust generation is expected to be spanwise uniform in these areas, yet the values vary by as much as 33% and do not agree within the bounds of the measurement uncertainty. This variability in ‘spanwise uniform’ thrust generation is noted and may be the result of variation in ambient humidity on different test days, known to affect actuator performance (Benard et al. 2009). Furthermore, the variability could also stem from imperfections introduced during actuator manufacturing, such as contamination and other minor defects arising during paint application and drying, since the variability extends beyond the limits of the measurement uncertainty. Nevertheless, the measurements are still capable of resolving the decrease in thrust generated as the edge of an active region is approached and passed for the spanwise modulated configurations (Fig. 9b).

Averaging $T_x$ within the active region (all measured locations for the two-dimensional configuration, and within $0 \le z \le$ 5 mm for the spanwise modulated configurations) gives the average thrust generated in the active region, $T_\text {act}$, which is plotted as a dashed line in Fig. 9 for each configuration. As expected, $T_\text {act}$ is highest for the two-dimensional configuration, which is not subject to any end effects, while $T_\text {act}$ is equal, within the experimental uncertainty, for the three spanwise modulated configurations. More sensitive experimental measurements are required to determine if the minimal decreases seen in $T_\text {act}$ with increasing spanwise wavelength are due to weakening jet interaction effects.

**Fig. 10:**
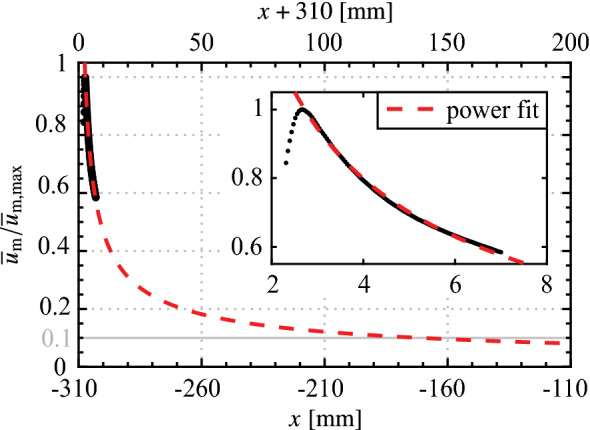
Streamwise decay of ${\overline{u}}_\text {m}$ for the 2D actuator wall-jet at *z* = 0 mm (from Fig. 8). Red dashed line is a power fit of the form ${\overline{u}}_\text {m} = Ax^\alpha$ from Narasimha et al. (1973)

Based on this quiescent characterization, it is of interest to assess the streamwise distance over which the spanwise modulated forcing can maintain an effective spanwise wavelength, since the diffusion of jet momentum will cause the disturbance front to eventually drop to some ineffectual level. This can be seen in Fig. 8, from which the locus of the local maxima of mean jet velocity, ${\overline{u}}_\text {m}$, is extracted and plotted in Fig. 10. The power law fit of Narasimha et al. (1973) for turbulent plane wall-jets is applied to the decaying portion of the profile. Extrapolating the fit, the jet is predicted to maintain at least 10% of its initial maximum velocity over a streamwise distance of 150 mm, covering 48% of the distance between the actuator and the mean separation point of the LSB (Fig. 5). As will be evaluated in Fig. 3.2, the intention is to activate spanwise instability modes that may be present in the boundary layer upstream of the laminar separation bubble (Rist and Augustin 2006), with the relatively slow decay of the forcing providing a significant streamwise distance over which to do so (Fig. 10).

**Fig. 11:**
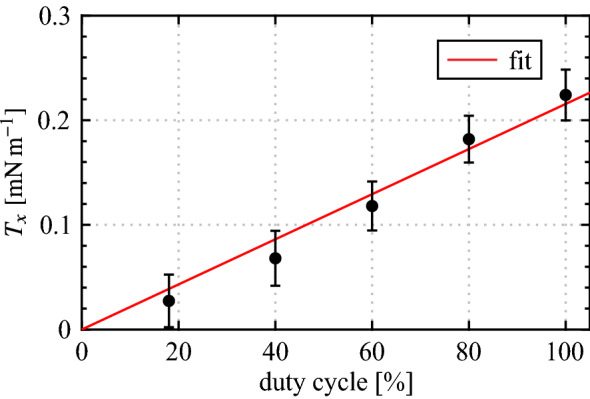
Effect of duty cycle on sectional thrust generated at *z* = 0 mm for the 2D actuator ($V_\text {pp}$ = 6 kV, $f_\text {c}$ = 5 kHz, $f_\text {m}$ = 133 Hz)

The results presented thus far pertain to operation at a duty cycle of 100% since, as discussed in Sect. 2, higher duty cycle operation leads to a more even distribution of seeding particles through increased turbulent mixing and therefore improved PIV measurements. Even so, the thrust estimates are subject to relatively high uncertainty (Fig. 9), with even higher uncertainties expected as the forcing duty cycle is decreased. In Sect. 3.2, the plasma actuators are operated at duty cycles between 21% and 25% at a modulation frequency of $f_\text {m}$ = 133 Hz in order to target the main instability in the LSB, and therefore a relationship between duty cycle and momentum output must be established. The result is presented in Fig. 11, showing thrust measured over a range of duty cycles for the two-dimensional configuration at the *z* = 0 mm plane. The uncertainty intervals remain approximately constant with duty cycle, confirming that results at lower duty cycles are subject to much higher relative uncertainty. Within the experimental uncertainty, the trend in $T_x$ with duty cycle is linear and thus the thrust generated for all configurations at other duty cycles is estimated through linear interpolation of the current results.

**Table 2:** Thrust and momentum coefficient estimates ($V_\text {pp}$ = 6 kV, $f_\text {c}$ = 5 kHz, $f_\text {m}$ = 133 Hz)

Configuration ($\lambda _{z}$)	Duty cycle [%]	$T_\text {tot}$ [$\upmu$N]	$C_\mu$ [$\times 10^{-4}$]	Superposition $C_\mu$ [$\times 10^{-4}$]
2D	100	$71.4 \pm 6.8$	$46.8 \pm 4.5$	–
25	100	$19.2 \pm 1.8$	$12.6 \pm 1.2$	–
37.5	100	$13.5 \pm 1.2$	$8.8 \pm 0.8$	–
50	100	$7.9 \pm 0.9$	$5.2 \pm 0.6$	–
2D	25	$18.6 \pm 3.8$	$12.2 \pm 2.4$	$12.2 \pm 2.4$
2D	21	$15.0 \pm 3.4$	$9.8 \pm 2.2$	$12.4 \pm 2.3$
25	21	$4.0 \pm 0.8$	$2.6 \pm 0.6$	
2D	22	$15.7 \pm 3.4$	$10.3 \pm 2.2$	$12.2 \pm 2.3$
37.5	22	$3.0 \pm 0.6$	$1.9 \pm 0.4$	
2D	23	$16.4 \pm 3.6$	$10.8 \pm 2.4$	$12.0 \pm 2.4$
50	23	$1.8 \pm 0.4$	$1.2 \pm 0.2$	

Since negligible thrust is produced at sections where the electrodes do not overlap (Figs. 7 and 9), estimates of the total thrust produced by an electrode pair can be made from the average sectional thrust generated in the active regions ($T_\text {act}$, Fig. 9) and the total active length, which are 385, 150, 110 and 70 mm for the 2D, $\lambda _{z}= 25$, 37.5, and 50 mm configurations, respectively. These results are presented in Table. 2, in terms of total thrust produced, $T_\text {tot}$, and momentum coefficients, $C_\mu = T_\text {tot}/\left( \frac{1}{2}\rho {u_\infty }^2\,l\delta ^*_\text {s}\right)$, where *l* is a configuration’s active length, and $\delta ^*_\text {s}$ = 2.0 mm is the displacement thickness at separation for the in-flow characterization (measured via PIV). As expected, total thrust production decreases with increasing spanwise wavelength as a result of decreasing active length.

The results in Table. 2 highlight that achieving equal momentum output across the four desired forcing scenarios is not possible by operating electrode pairs individually, since, for example, the $\lambda _{z}$ = 50 mm configuration at 100% duty cycle produces less than half the momentum of the two-dimensional configuration at 25% duty cycle. However, it is possible to achieve equal total momentum output by operating electrode pairs in tandem since the momentum output of multiple actuators in similar configurations has been shown to be linearly additive (Post and Corke 2004; Forte et al. 2007; Thomas et al. 2009). Thus, duty cycles for various actuator combinations can be identified that give equal amounts of total momentum output. These values are reported in Table. 2 under the superposition $C_\mu$ column, showing that the outputs of the $\lambda _{z}= 25$, 37.5, and 50 mm configurations combined with that of the two-dimensional configuration at duty cycles of 21%, 22%, and 23%, respectively, give superposition $C_\mu$ values that match the individual output of the two-dimensional configuration at a duty cycle of 25%. Thus, operating parameters for the two-dimensional forcing scenario and three cases of superposition with spanwise modulation have been identified. These parameters are expected to produce equivalent total momentum output across the four forcing scenarios and will be used for the in-flow characterization.

### In-flow characterization

The effort now shifts to in-flow characterization of the plasma forcing technique, where outputs are to be spatially superimposed to create three-dimensional disturbances of a prescribed spanwise wavelength. The flow considered is an LSB formed over a flat plate through an applied adverse pressure gradient (Figs. 4 and 5), chosen since previous findings suggest LSBs may have a dependence on incoming three-dimensional disturbances (e.g., Rist and Augustin 2006), which, if controlled, could have practical benefits, such as separation control and noise mitigation, as discussed in Sect. 1.

**Fig. 12:**
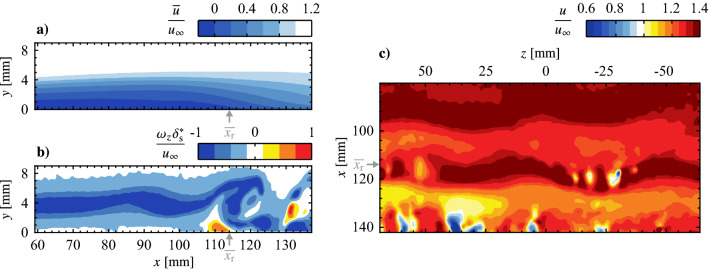
Flow field for in-flow characterization, in terms of **a** mean streamwise velocity, and representative instantaneous fields of **b** spanwise vorticity and **c** streamwise velocity. **a** and **b** are side PIV measurements at *z* = 0 mm; **c** is from top PIV measurements at *y* = 7 mm. Grey arrows mark the mean streamwise reattachment location ($\overline{x_\text {r}}$)

**Fig. 13:**
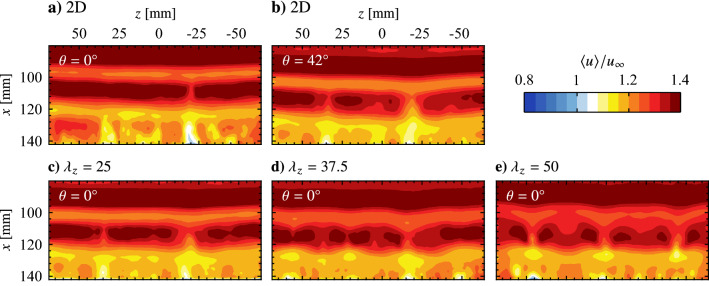
Phase-averaged streamwise velocity contours. Flow is from top-to-bottom. Note that the 2D actuator is off for **c**–**e**

The natural LSB flow field (no forcing) is presented in Fig. 12 in terms of the mean streamwise velocity field in the *z* = 0 mm plane (Fig. 12a) and two representative instantaneous fields showing the development of disturbances in the *z* = 0 mm and *y* = 7 mm planes (Figs. 12b and c, respectively). Figure 12a shows a typical mean streamwise velocity field in the aft portion of an LSB, as a small region of time-averaged reverse flow (darkest shade of blue) is present near the surface, indicating mean boundary layer separation and subsequent reattachment. The attendant shedding of shear layer vortices is captured in *x*-*y* and *x*-*z* planes in Figs. 12b and c, respectively. In Fig. 12c the vortices are marked by higher velocity contours with an approximate streamwise wavelength of $\lambda _{x}$ = 25 mm. Thus, the spanwise forcing wavelengths investigated here correspond to $\lambda _{z}= \lambda _{x}$, $1.5\lambda _{x}$ and $2\lambda _{x}$, which were intentionally selected to fall within the range reported for dominant spanwise undulations in naturally developing LSBs, $1 \le \lambda _{z}/\lambda _{x}\le 7$ (Marxen et al. 2013; Kurelek et al. 2016; Michelis et al. 2018, 2018a), with $2\lambda _{x}$ being the largest spanwise wavelength that could be accommodated given facility and measurement constraints. Indeed, notable spanwise undulations are observed in the natural LSB flow (Fig. 12c). It is this observation that points towards a possible secondary instability present within the LSB that amplifies spanwise disturbance modes, which in the case of the natural flow are initialized from the external disturbance environment. If this is the case, then such an instability can be investigated by the forcing technique developed herein and targeted for possible flow control applications.

**Fig. 14:**
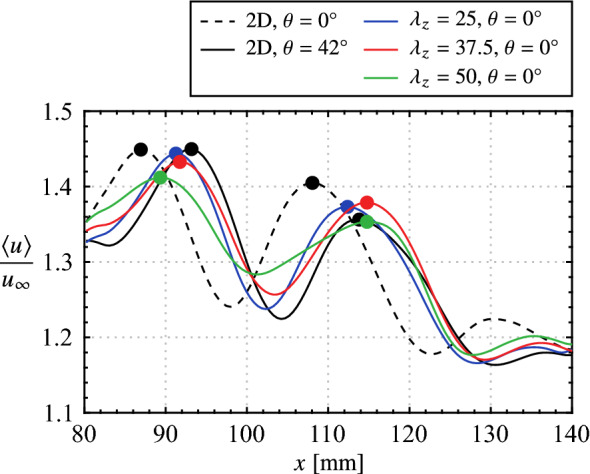
Phase-averaged streamwise velocity contours for phase $\theta = {0}^{\circ }$ showing disturbance superposition. Flow is from top-to-bottom

**Fig. 15:**
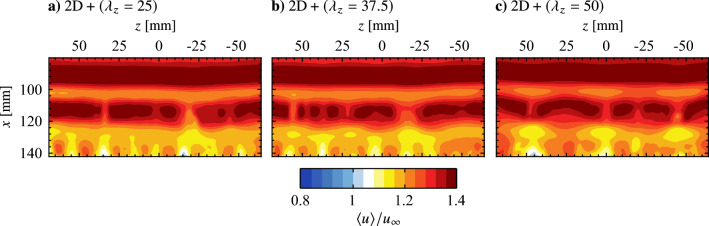
Phase and spanwise-averaged streamwise velocity for the same cases presented in Fig. 13. Markers (coloured according to legend) indicate selected maxima

An appropriate phase delay between the two-dimensional and spanwise modulated electrode pairs must be determined in order to spatially superimpose the produced disturbances. This phase delay is expected to depend on the spatial separation of the electrode pairs, the forcing parameters, and the external flow. To this end, PIV measurements synchronized to the forcing signals are acquired, with electrode pairs first operated in isolation. The results are presented in Fig. 13 in terms of phase-averaged streamwise velocity. Figures 13a and b show the progression of a forced two-dimensional disturbance (i.e., only the upstream electrode pair operating) at two different phases, while Fig. 13c–d show the flow field when only a spanwise modulated electrode pair is active. In all cases, two distinct spanwise vortices are captured in the field of view, marked by bands of relatively high streamwise velocity. When forcing is performed using only a spanwise modulated electrode pair (Figs. 13c–d), the structures are located at approximately equal streamwise locations ($x = 90$ and 112 mm) for the same phase angle in the forcing cycle. At the same phase angle, the vortices subject to the two-dimensional forcing (Fig. 13a) are captured farther upstream ($x = 86$ and 108 mm) compared to Fig. 13c–d, which is attributed to the upstream positioning of the two-dimensional electrode pair (Fig. 2). Advancing the phase by $42^\circ$ in the 2D forcing cycle (Fig. 13b) adjusts for this offset, which is verified in Fig. 15 via spanwise-averaged velocity data. The markers in Fig. 15 show local streamwise velocity maxima that estimate the streamwise position of the disturbance, which in turn allows for estimation of the optimal phase shift between the two-dimensional and spanwise modulated forcing. This phase shift is determined to be $42^\circ$ for all employed electrode arrangements.

The result of operating the electrode pairs in tandem, referred to as the $\text {2D} + (\lambda _{z}= 25)$, $\text {2D} + (\lambda _{z}= 37.5)$, and $\text {2D} + (\lambda _{z}= 50)$ cases, for disturbance superposition with the determined optimal phase delay is presented in Fig. 14. These three cases show the same streamwise disturbance wavelength, $\lambda _{x}\approx 25 \text {mm}$, as the two-dimensional forcing case (*cf*. Figure 13b). Thus, across these four forcing configurations, the produced disturbances are of the same frequency and streamwise wavelength. The former is the result of operating all actuators at the same modulation frequency ($f_\text {m}$ = 133 Hz), while the latter is achieved through the successful superposition of disturbances when the spanwise modulated actuators are active (Fig. 14).

**Fig. 16:**
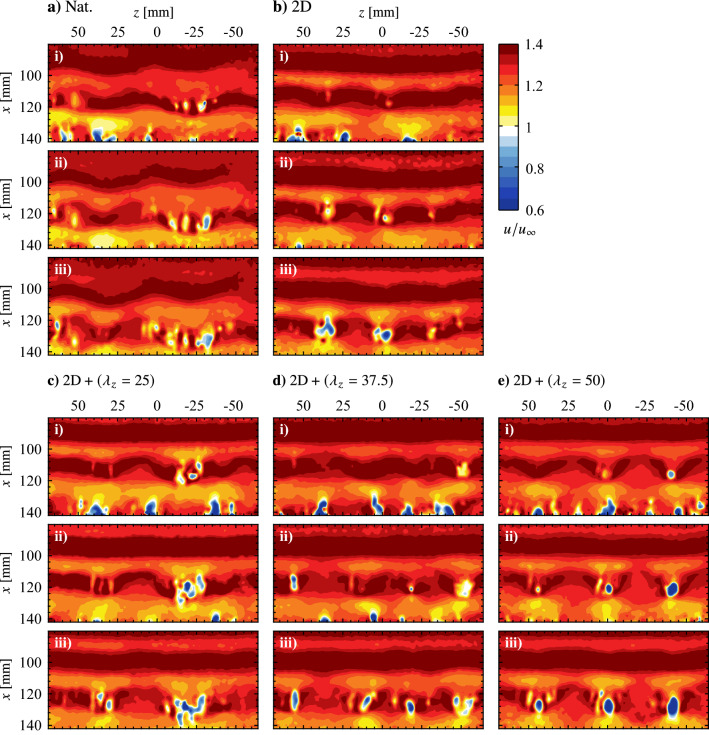
Sequences of instantaneous streamwise velocity contours. Flow is from top-to-bottom. Consecutive frames are separated by 1.71 ms. See supplementary material for accompanying videos (Online Resources 3–7, 10.1007/s00348-023-03616-9)

Flow development in the LSB is now examined to determine if the technique is successful in producing the desired spanwise disturbances. Figure 16 and accompanying supplementary videos (Online Resources 3–7, 10.1007/s00348-023-03616-9) present sequences of instantaneous streamwise velocity measured via PIV for the natural (unforced) flow and the four forcing configurations. As a point of comparison to the forcing cases, the predominant spanwise wavelengths in the naturally developing vortex filaments is of interest and is approximately $\lambda _{z}\,= 60\, \text {mm}$ for the sequence depicted in Fig. 16a. A rigorous statistical characterization follows later in this section. With a streamwise wavelength of $\lambda _{x}\approx 25\, \text {mm}$, the spanwise-to-streamwise wavelength ratio for the naturally developing structures in Fig. 16a is $\lambda _{z}/\lambda _{x}= 2.4$.

From Fig. 16, significant changes in flow development are observed when the flow is forced. Specifically, for the two-dimensional forcing case (Fig. 16b), the most upstream vortex in Fig. 16b–i is uniform across the span, and remains largely two-dimensional farther downstream while developing minor spanwise undulations, as seen at $x \approx 100$ mm in Fig. 16b–iii. These undulations are more apparent for the vortex located at $x \approx 115$ mm in Fig. 16b–i. The same vortex is located at $x \approx 120$ and 124 mm in Fig. 16b–ii and b–iii, respectively, and features localized breakup regions at $z \approx -35$, 0, and 35 mm where the filament surges forward in the streamwise direction. However, comparing the results in Figs. 16a and b at a given streamwise location (e.g., at $x = 120$ mm) reveals that the two-dimensional forcing leads to an increase in spanwise coherence in the formed shear layer vortices.

Similar to the results of the two-dimensional forcing case, highly spanwise uniform vortex filaments are observed upstream of $x = 100$ mm for all three spanwise modulated forcing cases (Figs. 16c–e), indicating that the disturbances introduced into the flow are primarily two-dimensional with only a weak spanwise component. The result is to be expected given that, from the quiescent characterization (Fig. 2), the two-dimensional forcing component provides 80 to 90% of the total momentum for these three cases. However, despite the relative weakness of the spanwise forcing components, spanwise wavelengths that match the forcing wavelength is evident for the $\text {2D} + (\lambda _{z}= 50)$ case (e.g., the structure at $x = 110$ mm in Fig. 16e–i), while the effect is not as apparent for the $\text {2D} + (\lambda _{z}= 25)$ and $\text {2D} + (\lambda _{z}= 37.5)$ cases (Figs. 16c and d, respectively). For the $\text {2D} + (\lambda _{z}= 50)$ case, note that the active elements of the spanwise modulated actuator are located at $z = -50$, 0, and 50 mm, while the vortex filaments in Fig. 16e lag behind at spanwise locations downstream of the actuator gaps.

**Fig. 17:**
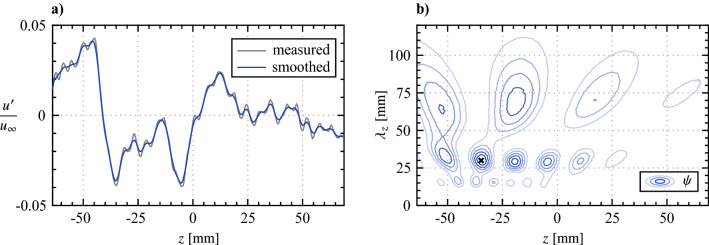
**a** Exemplary fluctuating streamwise velocity sampled across the span at *x* = 90 mm in the natural flow and **b** corresponding wavelet coefficient contours, $\psi$. Maximum wavelet coefficient indicated by $\varvec{\times }$ marker

The flow development depicted in Fig. 16e demonstrates that the forcing does not result in a simple spanwise modulation of the base flow. Rather, the fact that the undulations prescribed at a particular wavelength are amplified downstream points to the presence of a secondary instability mechanism within the LSB that amplifies the spanwise component of the input disturbances. Furthermore, the effect shows spanwise wavelength dependence, being most pronounced for the $\text {2D} + (\lambda _{z}= 50)$ case, while spanwise undulations matching the input wavelength of the $\text {2D} + (\lambda _{z}= 25)$ and $\text {2D} + (\lambda _{z}= 37.5)$ cases are not readily identified in the flow (Figs. 16c and 16d, respectively). Therefore, these cases may effectively force the flow in a manner more similar to the two-dimensional forcing, with evidence supporting this assertion following later in this section.

**Fig. 18:**
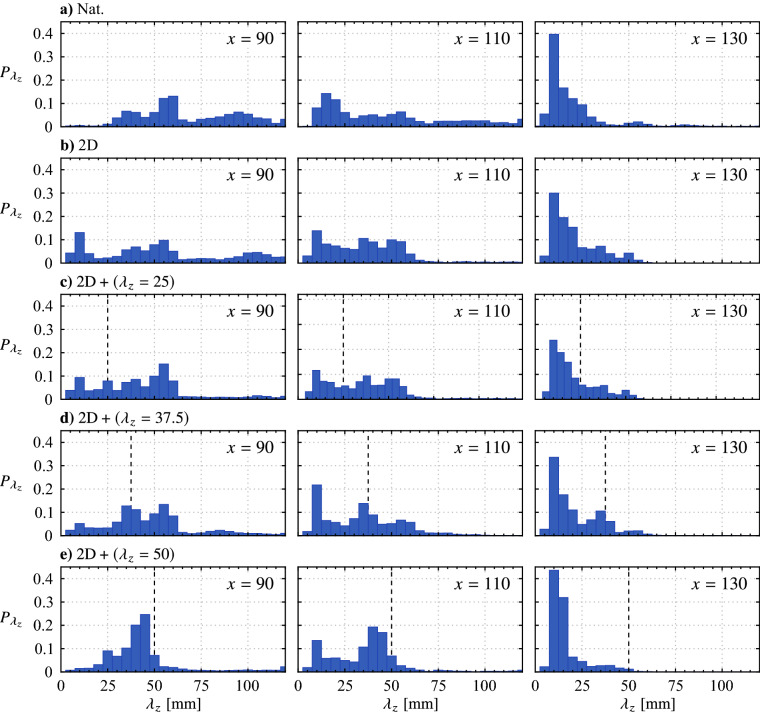
Spanwise wavelength probability distributions determined from spatial wavelet analysis (Fig. 17). Indicated *x* locations are given in mm. Black dashed lines in **c**–**e** mark the wavelength of the spanwise forcing component

Wavelet analysis is employed for statistical determination of the spanwise wavelengths that develop in the flow, which is well suited for the task since the field of view is limited to just over twice the largest spanwise forcing wavelength. From the top view PIV measurements (Fig. 16), fluctuating streamwise velocity signals are extracted at $x = 90$, 110 and 130 mm, smoothed using a 3 mm wide sliding spatial kernel, and wavelet coefficients, $\psi$, are calculated using the Morlet/Gabor wavelet (Daubechies 1992). An exemplary signal and its corresponding wavelet coefficients are presented in Fig. 17. The predominant spanwise wavelength for the given instant is estimated from the maximum wavelet coefficient ($\varvec{\times }$ marker in Fig. 17b), with the process repeated for all time realizations, resulting in 4480 statistical samples obtained per streamwise location, which are plotted as histograms in Fig. 18.

**Fig. 19:**
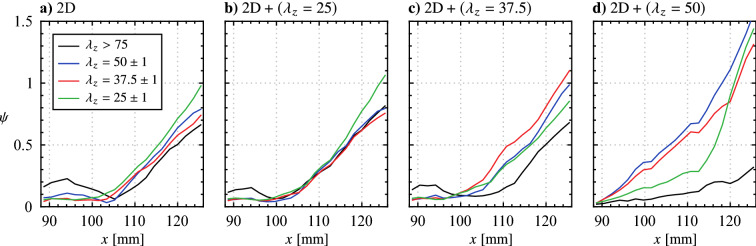
Streamwise growth of wavelet coefficients filtered to specific spanwise wavelength ranges (see legend). Coefficients are calculated from streamwise velocity signals sampled across the span of a vortex tracked through the phase-averaged PIV results (e.g., Figs. 13b and 14a–c)

For the natural case (Fig. 18a), the distribution of spanwise wavelengths at *x* = 90 mm is evenly spread across a wide range of wavelengths, $25 \lesssim \lambda _{z}\lesssim$ 120 mm, with a minor peak at $\lambda _{z}$ = 60 mm, roughly matching the characteristic wavelength of spanwise deformations seen in Fig. 16a at this location. The significant breadth of the histogram indicates that there is substantial variability in the spanwise undulations of the naturally developing shear layer vortices, with a minor preferences toward spanwise wavelengths of $\lambda _{z}\approx$ 60 mm. When compared to the predominant streamwise wavelength of the structures, $\lambda _{x}= 25$ mm, the general range of wavelengths reported at $x = 90$ mm in Fig. 18a is $1 \lesssim \lambda _{z}/\lambda _{x}\lesssim 5$, which is in agreement with results reported in previous investigations (Marxen et al. 2013; Kurelek et al. 2016; Michelis et al. 2018).

From Fig. 18a, the wavelength distributions shift to lower values as the vortices convect downstream to $x = 110$ and 130 mm, with a significant peak centred at $\lambda _{z}$ = 10 mm seen in the distribution of the most downstream station. This shift is associated with the turbulent breakup of the shear layer vortices, seen in Fig. 16a, as the prominent fluctuations that are localized to the breakup regions in the vortex filaments are characterized by these short wavelengths. In fact, all cases presented in Fig. 18 show this shift to shorter wavelengths as the flow develops downstream, confirming that this trend is indicative of turbulent breakdown.

The shear layer vortices are initially spanwise uniform when the flow is subjected to the two-dimensional forcing (Fig. 16b). Consequently the results at $x = 90$ mm in Fig. 18b show a wide distribution that spans the entire detectable range of wavelengths. Three minor peaks are detected in the distribution, two of which roughly match the natural case at the same streamwise location ($\lambda_{z} \approx 30$ and 100 mm). The peak at the lowest wavelength, $\lambda _{z}= 10$ mm, may indicate an intermittent early onset of turbulent breakdown, but could also result from noise in the PIV measurement that is not completely removed by the smoothing process.

It is instructive to begin examination of the spanwise modulated forcing results with the $\text {2D} + (\lambda _{z}= 50)$ case (Fig. 18e), where spanwise undulations at and near the forcing wavelength are detected in the flow at $x = 90$ mm. The peak in the histogram is found at $\lambda_{z}= 45$ mm which indicates there is a tendency for the flow to modify the forcing input wavelength as disturbances grow through the upstream boundary layer and into the LSB. It must be noted that this peak in the histogram is an indication of the tendency for undulations to develop at this wavelength and does not quantify their amplitude. Evaluations of the flow development (Fig. 16e) affirm that these undulations are of a relatively minor amplitude at this streamwise location, with a more rigorous quantification of spanwise disturbance amplitudes following this discussion. As the structures develop downstream to $x = 110$ and 130 mm, the distributions shift to lower wavelengths as a result of turbulent breakdown. A similar trend can be seen for the $\text {2D} + (\lambda _{z}= 37.5)$ case (Fig. 18d), as a minor peak centred at the forcing input wavelength is found in the $x = 90$ mm distribution, albeit the outcome is not as apparent as the $\text {2D} + (\lambda _{z}= 50)$ forcing. Thus, the forcing technique has revealed a wavelength dependence on three-dimensional disturbance growth for the given LSB flow, with growth at $\lambda _{z}\approx 50$ mm being preferred over $\lambda _{z}= 37.5$ and 25 mm. In fact, examining the results of the $\text {2D} + (\lambda _{z}= 25)$ forcing case (Fig. 18c) reveals no distributions with peaks at the forcing wavelength. Instead the histograms resemble those of the two-dimensional forcing case, indicating that the spanwise component introduced by this configuration has been damped out and the flow is primarily forced in a two-dimensional manner.

The observed wavelength dependence is further revealed through evaluation of the streamwise growth of wavelet coefficients filtered to specific spanwise wavelength ranges. Individual spanwise vortices are tracked throughout the domain of the phase-averaged PIV results (e.g., Figs. 13b and 14a–c), with streamwise velocity signals sampled through the core and across the span of the structures used to compute wavelet coefficients. The maximum wavelet coefficients within the ranges $\lambda _{z}> 75$, and $\lambda _{z}\in \left[ 49\ 51\right]$, $\left[ 36.5\ 38.5\right]$, and $\left[ 24\ 36\right]$ are then extracted for all available streamwise positions, which are plotted in Fig. 19 for the four forcing scenarios. The long wavelength range, $\lambda _{z}> 75$, is taken as a measure of two-dimensionality, which for the 2D forcing case (Fig. 19a) is the most energetic wavelength range upstream of $x = 100$ mm which is consistent with the flow development seen in Fig. 16b and the tendency for long wavelength disturbances reported at *x* = 90 mm in Fig. 18a. Moving downstream to $x \approx 105$ mm, the energy at the longest wavelengths dissipates while beginning to increase for all shorter wavelengths (50, 37.5 and 25 mm), which is indicative of the transition to turbulence as energy is redistributed from larger to smaller scales. Here, the most energetic wavelength range is $\lambda_{z}= 25\ \text{mm} \pm 1$, which was previously identified for all cases to be associated with localized breakup in the vortex filaments (Fig. 16), causing the shift to lower wavelengths in Fig. 18 with downstream development.

Similar trends in the wavelet coefficients are seen for the 2D and $\text {2D} + (\lambda _{z}= 25)$ cases (Figs. 19a and b), as the longest wavelengths are initially the most energetic, followed by a redistribution to shorter wavelengths led by $\lambda _{z}= 25$ mm. While the forcing in Fig. 19b does target this wavelength, there is no appreciable effect as the growth in shorter wavelength disturbances begins at approximately the same streamwise location ($x \approx 105$ mm) and attains similar amplitudes to the 2D case with downstream development. Thus, it is further reinforced that the $\text {2D} + (\lambda _{z}= 25)$ case primarily forces the flow in a two-dimensional manner. In contrast, the effects of the $\text {2D} + (\lambda _{z}= 37.5)$ and $\text {2D} + (\lambda _{z}= 50)$ forcing are apparent in Figs. 19c and d as, for the former, $\lambda _{z}= 37.5$ mm develops as the predominant wavelength following the decrease in initial two-dimensionality. This initiates at $x \approx 100$ mm which is farther upstream than where growth begins in the shorter wavelength for the previous two cases, while the effect is most significant for the $\text {2D} + (\lambda _{z}\,= 50)$ case as this growth begins upstream of $x = 90$ mm in Fig. 19d, with the region where $\lambda _{z}>$ 75 mm is most energetic likely falling upstream of the measurement domain. The flow’s tendency for preferential amplification of spanwise disturbances with $\lambda_{z}= 50$ mm is clear, as the corresponding wavelet coefficients grow rapidly with downstream development, attaining much higher amplitudes than any of the other three forcing cases. It is interesting to note that growth at $\lambda _{z}= 37.5$ mm closely follows that of $\lambda_{z} = 50$ mm in Fig. 19d, which may reflect the flow’s tendency to modify the input forcing wavelength to a slightly lower wavelength, as seen in Fig. 18e at $x = 110$ mm where the histogram peak is centred at $\lambda _{z}= 45$ mm. Thus, the most unstable spanwise wavelength for the given LSB is speculated to lie within $37.5 \le \lambda_{z} \le 50$ mm.

### Effects of forcing on the LSB

While the primary objective of this investigation is the development and characterization of the forcing technique, it is of interest to consider the effects of the various forcing configurations on the mean LSB characteristics since reducing the size of an LSB is a common flow control objective (e.g., Marxen and Henningson 2011; Yarusevych and Kotsonis 2017). For brevity, and given the more substantial effects of the $\text {2D} + (\lambda _{z}= 50)$ forcing case, the focus is placed on comparing results of this case with the natural flow and the two-dimensional forcing case.

**Fig. 20:**
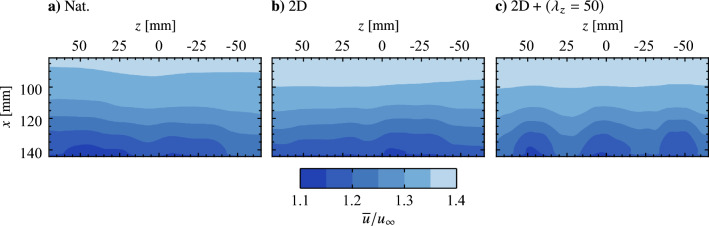
Contours of mean streamwise velocity at $y = 7$ mm. Flow is from top-to-bottom

**Fig. 21:**
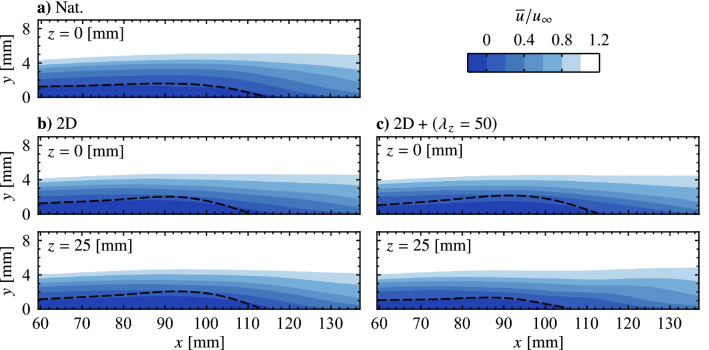
Contours of mean streamwise velocity at selected spanwise locations. Black dashed lines mark dividing streamlines, whose intersection with $y = 0$ mm estimates points of mean streamwise reattachment, ${\overline{x_\text {r}}}$

Insight into the spanwise, time-averaged spatial structure of the LSB is gained through examination of mean streamwise velocity in the $y = 7$ mm plane measured using the top PIV configuration. The results are presented in Fig. 20, where ${\overline{u}}$ at a fixed wall-normal height decreases significantly with increasing streamwise distance due to boundary layer separation and subsequent rapid growth in boundary layer thickness due to flow transition. Immediately apparent is a distinct spanwise non-uniformity present for the $\text {2D} + (\lambda _{z}= 50)$ case (Fig. 20c), in comparison to the relatively spanwise uniform flows present in natural conditions and under two-dimensional forcing (Figs. 20a and b, respectively). The latter is to be expected given the spanwise uniform static pressure distribution (Fig. 5b) and the nature of the 2D forcing, while the flow pattern seen in Fig. 20c exhibits a distinct spanwise wavelength of $\lambda_{z} = 50$ mm, indicating that the forcing has affected the mean spatial structure of the LSB in a spanwise non-uniform manner.

This is further examined through the side PIV results at select spanwise locations (*z* = 0 and 25 mm for the forcing cases, *z* = 0 mm only for the natural case) presented in Fig. 21, where the LSB is characterized using the mean dividing streamline, which is found as the streamline that forms a closed contour with the surface within which the streamwise mass flux is zero (O’Meara and Mueller 1987). The intersection point of the dividing streamline with the surface is used to estimate the mean streamwise reattachment point, ${\overline{x_\text {r}}}$. The results in Fig. 21 show the natural LSB reattaches, in the mean sense, at ${\overline{x_\text {r}}}$ = 114.0 mm at *z*

= 0 mm.

**Fig. 22:**
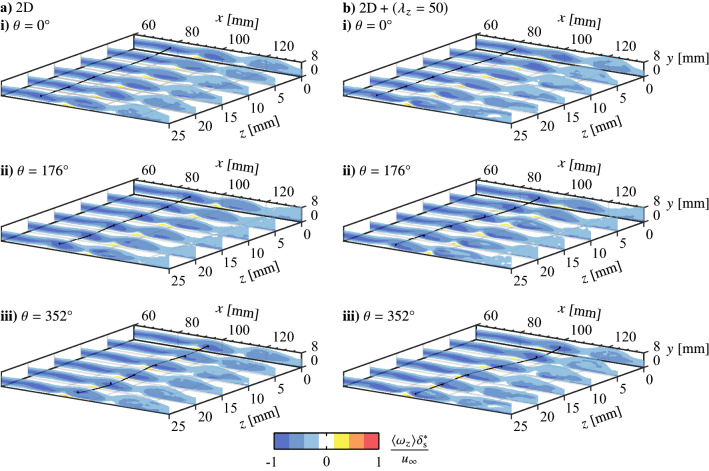
Sequence of phase-averaged spanwise vorticity contours. Circle markers indicate vorticity cores for a given structure, identified via the $\lambda _2$-criterion (Jeong and Hussain 1995), and are connected using smoothing spline fits

The spanwise effects of the $\text {2D} + (\lambda _{z}= 50)$ forcing case are most readily apparent through comparison with the 2D forcing case at *z* = 25 mm in Fig. 21b and c, where significant differences are observed. Notably, the $\text {2D} + (\lambda _{z}= 50)$ forcing causes a significant reduction in the wall-normal height of the mean dividing streamline and an upstream shift of ${\overline{x_\text {r}}}$ to 105.4 mm, compared to ${\overline{x_\text {r}}}$ = 113.3 mm for the 2D case at the same plane and ${\overline{x_\text {r}}}$ = 114.0 mm for the natural case at *z* = 0 mm. Thus, the $\text {2D} + (\lambda _{z}= 50)$ forcing results in a notable reduction in the local size of the LSB, leading to the spanwise-distorted spatial structure seen in Fig. 20c. Note that *z* = 25 mm sits halfway between the active elements of the $\text {2D} + (\lambda _{z}= 50)$ actuator and, per Fig. 16e, is where the shear layer vortices were found to lag behind in the streamwise direction, while surging forward at $z = -50$, 0 and 50 mm. Thus, in tandem with Fig. 21c, the results indicate that the effects of the spanwise modulated forcing on the mean LSB characteristics are most pronounced in regions of the streamwise rearward ‘valleys’ of the shear layer vortices (*z* = 25 mm), while in the ‘crest’ regions (*z* = 0 mm), the forcing effects are similar to that of the 2D case. Thus, inline with previous findings demonstrating that the time-averaged shape and size of an LSB are most effectively modified by gaining control authority over the shear layer vortices (Marxen and Henningson 2011; Yarusevych and Kotsonis 2017), here, it is demonstrated that the mean spanwise spatial structure of the LSB can be altered significantly (e.g., Fig. 20) through control of the shear layer vortices and their spanwise characteristics.

To reinforce the influence of the shear layer vortices on the mean LSB structure, Fig. 22 presents contours of phase-averaged spanwise vorticity measured at several *x*-*y* planes using the side PIV configuration. Results are only presented for the 2D and $\text {2D} + (\lambda _{z}= 50)$ as phase-averaging is not possible for the natural case. To aid in visualizing the development of the shear layer vortices, the cores of vorticity of selected structures are identified using the $\lambda _2$-criterion (Jeong and Hussain 1995), which are then connected using smoothing spline fits. The results in Fig. 22 affirm the observation from the top PIV measurements (Figs. 16b and e), as the vortex filaments are initially two-dimensional under both types of forcing. As the vortices develop downstream, a clear spanwise undulation matching the forcing wavelength develops for the $\text {2D} + (\lambda _{z}= 50)$ case (Fig. 22b–iii), that is absent in the presence of the purely two-dimensional forcing (Fig. 22a–iii). Figure. 22b–iii also reveals that the core of the shear layer vortex at $x \approx$ 100 mm (marked by the spline fit) is closer to the surface in the region $z \ge$ 15 mm, which is where the filament lags behind the streamwise forward section that develops within $z \le$ 10 mm. Ultimately, these filament motions correlate with the changes seen in the mean characteristics in Fig. 21c, as the vortex filament moves closer to the surface at *z* = 25 mm, aligning with the spanwise regions where the mean streamwise and wall-normal extents of the LSB are reduced.

## Conclusions

In this investigation, a three-dimensional forcing technique capable of producing deterministic disturbances modulated to a desired spanwise wavelength was developed and characterized, alongside an exemplary implementation to a laminar separation bubble flow. The technique utilized AC-DBD plasma actuators, with two sets of exposed and covered electrodes installed on a single dielectric layer and arranged in streamwise succession. Two-dimensional forcing was achieved through isolated operation of the upstream, spanwise uniform electrode pair, while three-dimensional forcing at a prescribed spanwise wavelength was attained by operating both pairs simultaneously. The upstream pair produced a spanwise uniform disturbance, which was then spanwise modulated by the downstream pair, with a phase delay used to superimpose the two outputs.

A detailed characterization of the actuators was carried out in both quiescent and in-flow conditions to determine the spanwise and streamwise disturbance characteristics, total momentum output, and phase delay needed for disturbance superposition. The quiescent characterization involved flow visualizations and planar PIV measurements, showing that all configurations provide a spanwise uniform injection of streamwise momentum through the production of a relatively weak, wall-parallel streamwise jets in regions where a covered and ground electrode overlap. Thus, two-dimensional output is achieved from a configuration with electrode overlap that is spanwise continuous, while disturbances of a desired spanwise wavelength are produced through introducing gaps in the electrode overlap, as no streamwise velocity is generated in the gap regions.

From the planar PIV measurements, the generated thrust and associated momentum coefficients were estimated for all electrode pairs using a control volume analysis. The results highlight higher sectional thrust generated by the two-dimensional configuration as end effects decrease momentum output at the edges of the overlap regions of the spanwise segmented actuators. This, coupled with different total lengths of the regions in which thrust is produced, results in varied total thrust production for all the electrode pairs considered. Through assumed superposition of momentum output for pairs arranged in streamwise succession and an established linear trend between thrust output and forcing duty cycle, forcing parameters that yield equal amounts of total momentum output were identified for the desired forcing scenarios. In particular, duty cycles of 25%, 21%, 22% and 23% were found to give equal total momentum across the 2D, $\text {2D} + (\lambda _{z}= 25)$, $\text {2D} + (\lambda _{z}= 37.5)$, and $\text {2D} + (\lambda _{z}= 50)$ cases, respectively, while all other forcing parameters were kept constant. The phase delay required to superimpose the disturbance outputs of the actuators was invariant to spanwise wavelength, with a value of $42^\circ$ providing successful disturbance superposition for the in-flow conditions considered.

Use of the technique in an exemplary application, a laminar separation bubble formed over a flat plate, was explored, with this particular flow configuration chosen since previous findings suggest LSBs may be sensitive to incoming spanwise disturbances (Rist and Augustin 2006). Through assessment of the flow development and a statistical characterization of spanwise disturbance wavelengths present in the flow, the results indicated that the spanwise forcing technique did not simply result in a spanwise modulation of the base flow, but rather revealed preferential amplification of perturbations at particular spanwise wavelengths. For the flow and parameters considered here, the amplification of spanwise undulations was most pronounced for a spanwise wavelength that was approximately double the streamwise wavelength of structures. The effect of shorter spanwise wavelength forcing was less apparent, matching more closely with the effects of the two-dimensional forcing.

This investigation serves as a proof-of-concept for producing three-dimensional disturbances of a desired spanwise wavelength via disturbance superposition of AC-DBD plasma actuator outputs. The technique could have immediate practical benefits for experimentally investigating spanwise flow instabilities in wall-bounded shear flows, much like the vibrating ribbon technique in the seminal work of Klebanoff et al. (1962). Further, the approach can offer a new degree of control authority in flow control application, for example in separating-reattaching flows, such as LSBs, where reducing spanwise coherence shear layer vortices can help mitigate airfoil tonal noise (Brooks et al. 1989).

## Electronic supplementary material

Below is the link to the electronic supplementary material.

Online Resource 1 (MP4 29078 kb)

Online Resource 2 (MP4 22998 kb)

Online Resource 3 (MP4 3022 kb)

Online Resource 4 (MP4 2812 kb)

Online Resource 5 (MP4 2814 kb)

Online Resource 6 (MP4 2884 kb)

Online Resource 7 (MP4 2911 kb)
